# Myocardial Infarction in Conduction System Pacing: Trust the ECG or Rely on Algorithms?

**DOI:** 10.1111/pace.70068

**Published:** 2025-11-18

**Authors:** Elena Crisman, Alexandra‐Maria Neagoe, Andreas Haeberlin, Jonas Häner, Bruno Schnegg

**Affiliations:** ^1^ Department of Cardiology, Inselspital, Bern University Hospital University of Bern Bern Switzerland

**Keywords:** conduction system pacing, electrocardiogram interpretation, myocardial ischemia Sgarbossa criteria, ST‐elevation myocardial infarction

## Abstract

A 66‐year‐old male patient with a conduction system pacing (CSP) pacemaker, implanted after a pace‐and‐ablate strategy for persistent, poorly rate‐controlled atrial fibrillation, presented to the outpatient clinic with worsening dyspnea. Laboratory findings and ECG were indicative of an anterolateral ST‐segment elevation myocardial infarction (STEMI), confirmed by coronary angiography showing occlusion of a large diagonal branch. STEMI diagnosis in paced patients is often challenging due to altered ventricular activation. However, this case demonstrates that surface ECG remains interpretable with CSP, which preserves physiological activation, allowing accurate localization of ischemia even in the presence of a pacemaker.

**Abbreviations**
AMI: acute myocardial infarction
CSP: conduction system pacing
HBP: his bundle pacing
LAD: left anterior descending
LBBAP: left bundle branch area pacing
LCx: left circumflex artery
OSC: original Sgarbossa criteria

## 1 Case Report

A 66‐year‐old man with heart failure with mildly reduced ejection fraction (HFmrEF, EF 45%) presented for routine outpatient cardiology follow‐up.

He had hypertensive heart disease for many years, worsened by permanent atrial fibrillation. Two years earlier, a cardiac function deterioration due to poor rate control (LVEF 30%) led to a “pace‐and‐ablate” strategy with left bundle branch area pacing (LBBAP). Three months later, LVEF had improved to 45%. Coronary angiography ruled out an ischemic component, revealing ectatic coronary arteries with significant atherosclerosis, but no critical stenosis.

**FIGURE 1:**
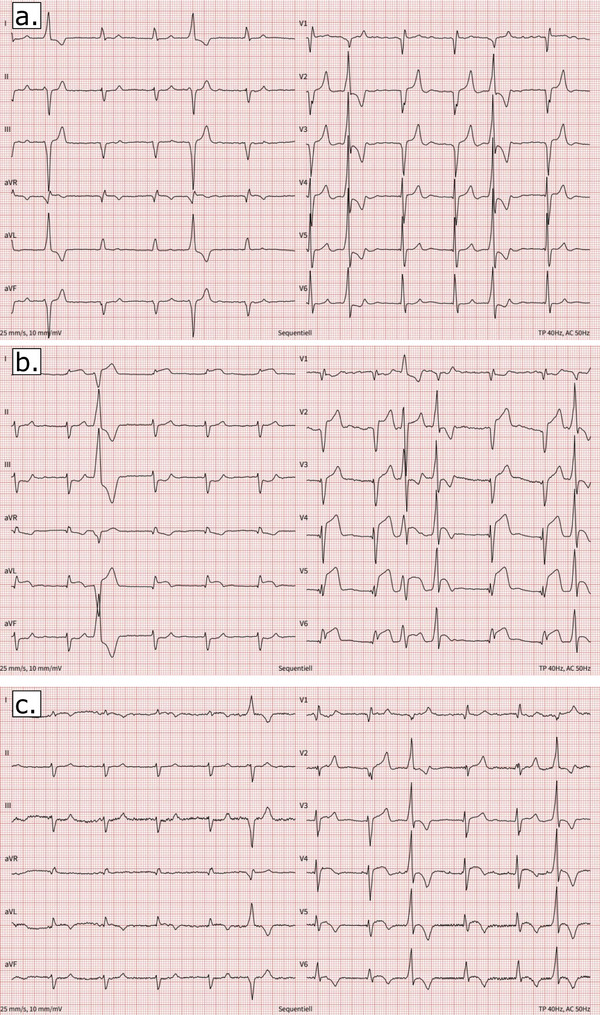
(a) Top panel (1 year prior): Baseline ECG showing atrial fibrillation with a paced QRS complex, without ST‐segment abnormalities. (b) Middle panel (day of event): Subacute anterior STEMI with marked ST elevations in leads V2–V6 and I, aVL, and reciprocal ST depressions in III and aVF. (c) Bottom panel (2 months post‐event): ECG shows resolution of ST elevations with post‐infarction T‐wave inversions in the anterolateral leads, consistent with myocardial recovery. [Colour figure can be viewed at wileyonlinelibrary.com]

He reported worsening general condition and exertional dyspnea, but no chest pain. The ECG showed atrial fibrillation with permanent ventricular pacing, marked ST‐segment elevations in the anterior and lateral leads, and ST depressions in leads III and aVF, which were absent in a recording performed 1 year earlier (Figure 1).

Echocardiography revealed new severely impaired left ventricular systolic function with EF of 30% with newly apical dyskinesia. Troponin‐T‐hs and NT‐proBNP had significantly increased (Troponin‐T‐hs: 4800 ng/L; baseline 30 ng/L; reference <14 ng/L; NT‐proBNP: 29,700 pg/mL; baseline 7400 pg/mL; reference <490 pg/mL).

**FIGURE 2:**
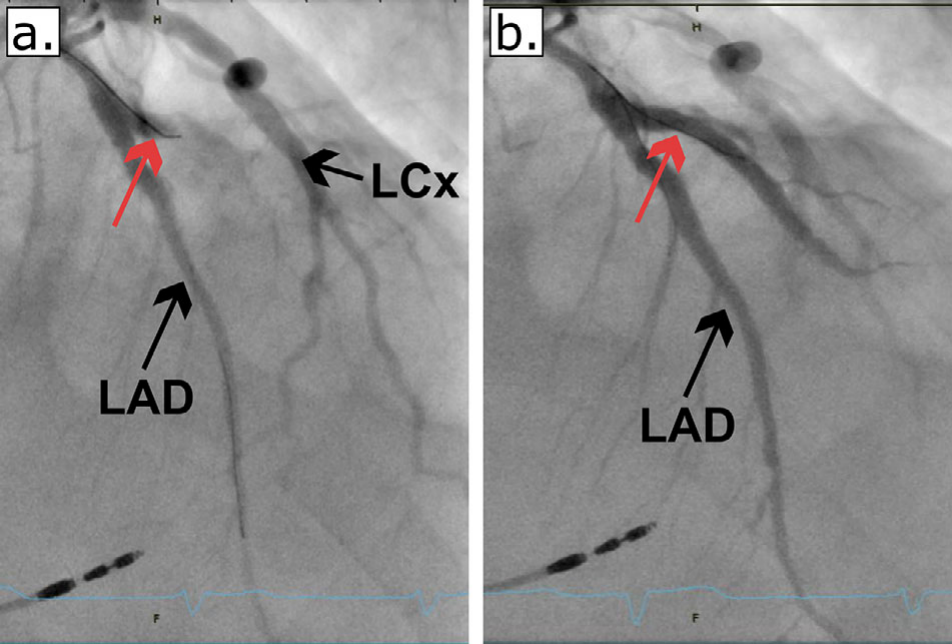
(a) (pre‐intervention): Coronary angiography in the left anterior oblique (LAO) cranial projection demonstrates an occlusion of the large diagonal branch (red arrow), consistent with the ECG changes and regional wall motion abnormalities. (b) (post‐intervention): Following successful percutaneous coronary intervention (PCI) with drug‐eluting stent implantation, the previously occluded diagonal branch shows restored TIMI III flow, confirming successful revascularization. [Colour figure can be viewed at wileyonlinelibrary.com]

A subacute STEMI was suspected, and prompt coronary angiography confirmed a subacute occlusion of a large ectatic diagonal branch, consistent with the ECG findings. The lesion was successfully recanalized and treated with a drug‐eluting stent, restoring TIMI III flow in the diagonal branch (Figure 2).

Post‐procedurally, the patient remained asymptomatic and hemodynamically stable. The troponin levels gradually decreased, and the ST‐segment elevations showed a regressive profile during the first 48 h. Following stent implantation, the patient was treated with aspirin, clopidogrel, and apixaban for 1 month, then clopidogrel and apixaban for 12 months, and continued on apixaban long‐term. Heart failure therapy was kept at the highest tolerated dose. Two months after this episode, the ST segment had completely normalized, and T wave inversions were noted as post‐infarct changes (Figure 1). Echocardiography performed 2 months after revascularization demonstrated persistent left ventricular dysfunction, with no improvement in LVEF despite guideline‐directed heart failure therapy.

## 2 Discussion

The ECG diagnosis of occlusion myocardial infarction in pacemaker patients is both challenging and essential for ensuring timely reperfusion therapy [1]. This difficulty stems from the non‐physiological ventricular depolarization, which results in abnormal repolarization, making the application of standard repolarization‐based diagnostic criteria unreliable. Consequently, this often leads to delays in treatment, with these patients facing a mortality rate twice as high as those without pacemakers [2].

The current guidelines suggest using the original Sgarbossa criteria (OSC) for diagnosing myocardial infarction in patients with ventricular‐paced rhythm, similar to those with left bundle branch block. While OSC has high specificity (90%), its low sensitivity (56%) would lead to non‐detection of 1 in 2 occlusive myocardial infarctions. Guidelines also recommend temporarily switching off the pacemaker to assess for classical ischemic ST‐segment changes, though interpretation can be confounded by potential stimulation‐induced changes (cardiac memory) [3]. However, this is not feasible in pacemaker‐dependent or unstable patients.

The Smith‐modified Sgarbossa criteria offer improved sensitivity (81%) with good specificity (84%) [4], yet the risk of delayed diagnosis and treatment in patients with right ventricular pacing remains elevated.

Conduction system pacing (CSP) strategies are increasingly adopted by implanters. By directly stimulating the patient's conduction system—either the His bundle (His bundle pacing, HBP) or the left bundle branch via a trans‐septal approach (LBBAP)—CSP is characterized by a more physiological ventricular activation as compared to traditional pacing methods. CSP may reduce mortality and heart failure hospitalizations compared to right ventricle apical pacing [5] and potentially even cardiac resynchronization therapy [6]. However, it is not well known how this more physiologic myocardial depolarization impacts the diagnosis of myocardial infarction. The Sgarbossa criteria and other related algorithms were developed for patients with left bundle branch block (LBBB) and ventricular pacing, where the QRS morphology closely mimics that of LBBB, but not for CSP.

LBBAP has become the predominantly used CSP modality due to the larger target area for lead implantation and the excellent electrical electrode properties. While only selective His‐Bundle pacing would result in a completely natural ventricular activation, and part of the ventricular activation is still mediated by propagation of the activation wavefront through working myocardium, LBBAP results in relatively narrow QRS complexes. LBBA‐paced QRS complexes typically show (incomplete) right bundle branch morphology and often almost “normal” repolarization (Figure 1, top panel). Thus, it has the potential to facilitate ECG interpretation in the setting of suspected myocardial ischemia.

As the technique is increasingly used, a better understanding of myocardial electrophysiological behavior during ischemia and infarction in LBBAP patients is of utmost importance to allow early diagnosis and timely treatment. To date, current evidence is limited to a few case reports [7, 8], and guidelines do not provide specific diagnostic recommendations.

The present case shows (1) that in an LBBAP patient, myocardial infarction due to the occlusion of a large diagonal branch resulted in anterolateral ST‐segment elevation comparable to that seen in patients with native conduction, allowing immediate diagnosis and primary PCI, and (2) that post‐infarction electrocardiographic changes were similar to those in non‐paced patients. Hence, these findings suggest that LBBAP preserves near‐physiological ventricular activation and repolarization not only at rest, but also in the setting of acute myocardial infarction.

This case, together with previously published experience [7, 8, 9], highlights three key considerations in patients with LBBAP: first, ST‐segment deviations should not be regarded as nonspecific, given that LBBAP produces a near‐normal QRS morphology. Second, ST‐segment alterations may offer diagnostic accuracy similar to that observed in the native rhythm ECG. Finally, LBBAP and overall CSP, by preserving physiological cardiac activation, may enable faster diagnosis and treatment in acute occlusion myocardial infarction, compared to traditional pacing systems.

## 3 Conclusion

Conduction system pacing facilitates the diagnosis of acute myocardial infarction in permanently paced patients. Moreover, CSP allows identifying accurate infarct localization on the ECG, unlike conventional pacemakers.

## Funding

The author received no specific funding for this work.

## Disclosure

The authors have nothing to report.

## Consent

The patient provided informed consent to submit this case for publication.
